# The multiple sex chromosomes of platypus and echidna are not completely identical and several share homology with the avian Z

**DOI:** 10.1186/gb-2007-8-11-r243

**Published:** 2007-11-16

**Authors:** Willem Rens, Patricia CM O'Brien, Frank Grützner, Oliver Clarke, Daria Graphodatskaya, Enkhjargal Tsend-Ayush, Vladimir A Trifonov, Helen Skelton, Mary C Wallis, Steve Johnston, Frederic Veyrunes, Jennifer AM Graves, Malcolm A Ferguson-Smith

**Affiliations:** 1 Department of Veterinary Medicine, University of Cambridge, Madingley Road, Cambridge CB3 OES, UK; 2 School of Molecular and Biomedical Science, The University of Adelaide, Adelaide, 5005 SA, Australia; 3 Institute of Cytology and Genetics, Russian Academy of Sciences, Siberian Department, 630090, Novosobirsk, Russia; 4 School of Animal Sciences, Currumbin Sanctuary, Queensland University, Brisbane, QLD 4072, Australia; 5 Research School of Biological Sciences, The Australian National University, Canberra, ACT 2601, Australia

## Abstract

**Background:**

Sex-determining systems have evolved independently in vertebrates. Placental mammals and marsupials have an XY system, birds have a ZW system. Reptiles and amphibians have different systems, including temperature-dependent sex determination, and XY and ZW systems that differ in origin from birds and placental mammals. Monotremes diverged early in mammalian evolution, just after the mammalian clade diverged from the sauropsid clade. Our previous studies showed that male platypus has five X and five Y chromosomes, no SRY, and DMRT1 on an X chromosome. In order to investigate monotreme sex chromosome evolution, we performed a comparative study of platypus and echidna by chromosome painting and comparative gene mapping.

**Results:**

Chromosome painting reveals a meiotic chain of nine sex chromosomes in the male echidna and establishes their order in the chain. Two of those differ from those in the platypus, three of the platypus sex chromosomes differ from those of the echidna and the order of several chromosomes is rearranged. Comparative gene mapping shows that, in addition to bird autosome regions, regions of bird Z chromosomes are homologous to regions in four platypus X chromosomes, that is, X_1_, X_2_, X_3_, X_5_, and in chromosome Y_1_.

**Conclusion:**

Monotreme sex chromosomes are easiest to explain on the hypothesis that autosomes were added sequentially to the translocation chain, with the final additions after platypus and echidna divergence. Genome sequencing and contig anchoring show no homology yet between platypus and therian Xs; thus, monotremes have a unique XY sex chromosome system that shares some homology with the avian Z.

## Abstract 2

A comparative study of the karyotype of the short-beaked echidna shows that monotremes appear to have a unique XY sex chromosome system that shares some homology with the avian Z.

## Background

Monotreme mammals are receiving increasing attention in genomic research, with interests varying from karyotype evolution and gene mapping, to comparative sequencing. This should not come as a surprise, as monotremes (mammalian Subclass Prototheria) occupy a unique branch at the base of the mammalian phylogenetic tree, and serve as an evolutionary outgroup for marsupial and eutherian species (that together comprise Subclass Theria). The time of divergence of Prototheria and Theria is estimated to be in the Early Jurassic (166 million years ago (MYA)), while marsupials and eutherians diverged in the Late Jurassic (148 MYA) [1]. Five extant monotreme species are recognized; platypus (*Ornithorhynchus anatinus*), short-beaked echidna (*Tachyglossus aculeatus*) and three long-beaked echidnas (*Zaglossus bruneiji*, *Zaglossus attenboroughi*, *Zaglossus bartoni*). *Zaglossus bartoni *is divided into three subspecies *Z. b. smeenki*, *Z. b. diamondi*, and *Z. b. cluniu*s [2].

A full karyotype characterization is essential for genomic research in any species. It is particularly important for monotremes because of their exceptional sex chromosome complement. The inclusion of a set of tiny chromosomes was recognized early and thought to be a reptilian feature [3], but this suggestion was later refuted [4]. A surprise was the discovery of several unpaired chromosomes [5]. A final identification and description of the platypus unpaired chromosomes was achieved only recently by our chromosome painting studies [6,7]. The 21 autosome pairs were assigned by chromosome paints. Ten paints identified ten unpaired mitotic chromosomes as well as the ten members of the meiotic chain and the homologous regions between them. Five paints identified X chromosomes present in single copy in males but two copies in females, and five paints identified Y chromosomes that were present only in males. It was, therefore, concluded that the ten male unpaired chromosomes consisted of five X and five Y sex chromosomes. The ten sex chromosomes form a multivalent chain at meiosis held together by chiasmata within homologous pairing regions. Alternate segregation of these chromosomes into X_1_X_2_X_3_X_4_X_5 _and Y_1_Y_2_Y_3_Y_4_Y_5 _sperm was proposed and must be very efficient as shown by meiotic analysis of spermatids and sperm using the paint probes [6]. Remarkably, X_5 _shows some homology with the chicken Z, as demonstrated by its inclusion of the *DMRT-1*, *DMRT-2 *and *DMRT-3 *orthologues [6,8]. Chicken Z is largely homologous to parts of human chromosomes 5 and 9, with some genes represented on 8 and 18 [9]. A region containing *ATRX*, *RBMX *and genes flanking *XIST*, present on Xq in human and other therians, maps to chromosome 6 in platypus [10], as does *SOX3*, the gene from which the sex-determining *SRY *gene evolved (M Wallis, personal communication), and this is consistent with the absence of a platypus homologue of the Y-linked *SRY*. Other genes involved in the eutherian sex determining pathway have recently been mapped to platypus autosomes, so do not qualify as candidate primary sex determining genes [11]. There is no platypus homologue of the human X-borne *XIST *in platypus [12] and marsupials [13]. In addition, platypus Ensembl release 44 and separate mapping work (F Veyrunes, personal communication) show an absence of human X-linked orthologues from platypus X_- _chromosomes, contradicting original localizations using radioactive fluorescent *in situ *hybridization (FISH) with heterologous cDNA probes [14-18]. It follows that *SRY *and the therian XY sex determining system have evolved between 166 and 148 MYA after the divergence of monotremes and before the divergence of marsupials, which is being explored further (F Veyrunes, personal communication).

To provide new clues to the organization, function and evolution of the platypus multiple sex chromosomes, we defined the sex chromosomes of the distantly related short-beaked echidna, *T. aculeatus*, and established the sex chromosome order in the echidna multivalent chain. Our genome-wide comparison using chromosome painting between echidna and platypus (called *Tac *(for *T. aculeatus*) and *Oan *(for *O. anatinus*) in this report) showed, surprisingly, that one member of the *Oan *chain is replaced by an autosome in *Tac*, and the X homologous to *Oan *X_5 _occupies a central position in the *Tac *chain rather than a position at the end as seen in *Oan*. To investigate the participation of the ancestral avian Z in the evolution of the monotreme sex chromosome system and to map genes to the members of the sex chromosomes, we also localized the platypus homologues of genes on chicken autosomes and Z. We conclude that the ancestral monotreme sex chromosome system bears considerable homology to the sex chromosomes of birds.

## Results

### Characterization of the short-beaked echidna karyotype

**Figure 1:**
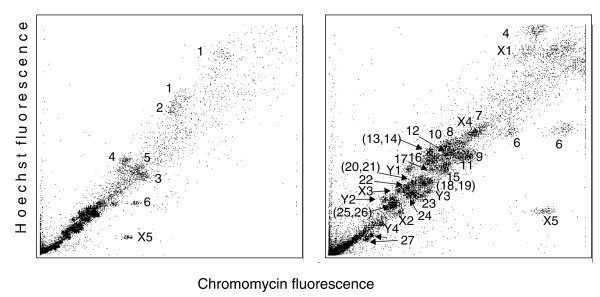
Flow karyotype of *T. aculeatus*. The left panel shows the upper part and the right panel the lower enlarged part of the flow karyotype. The following chromosomes sort together: 13,14; 18,19; 20,21; and 25,26. Chromosomes 1, 6, and 27 each are polymorphic and are represented by two peaks.

The male short-beaked echidna has 63 chromosomes and the female 64 [5]. We characterized and classified the male karyotype by flow cytometric analysis, flow sorting, and chromosome painting. The chromosomes produced 36 peaks in the flow karyotype (Figure 1); 17 peaks represent single chromosome pairs and 4 peaks represent 2 chromosome pairs each. The homologues of chromosomes 1, 6, 16, and 27 (which are frequently heteromorphic) each sort in two different peaks. The nine remaining peaks represent single chromosomes, which we show to be the nine unpaired sex-chromosomes that constitute the meiotic chain in the male echidna. Thus, the male echidna has 27 autosome pairs and 9 sex chromosomes.

**Figure 2:**
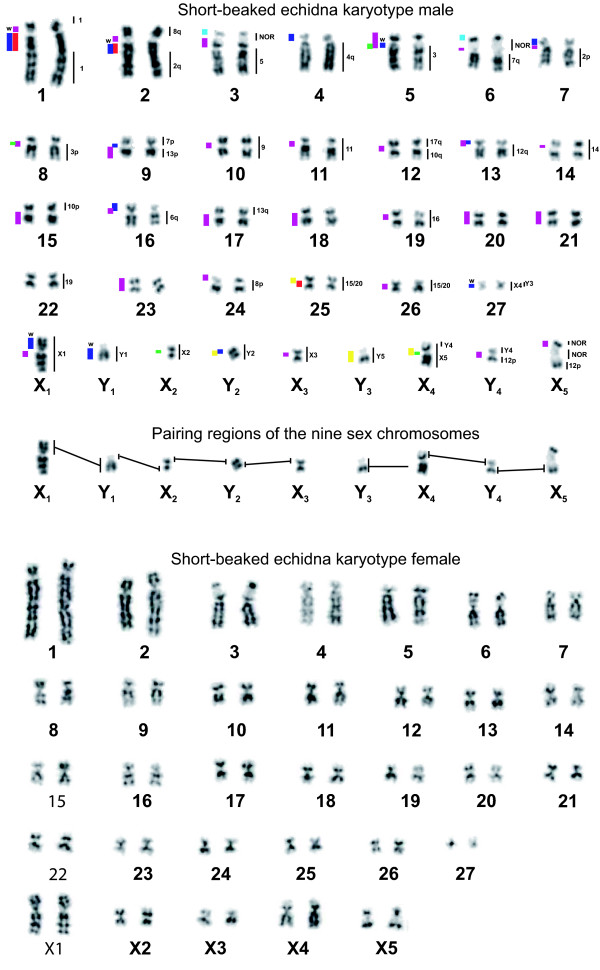
G-banded karyotype of *T. aculeatus*. Top: the male has 27 chromosome pairs and 9 unpaired chromosomes. Three kinds of information are given next to the chromosomes. Chromosomes 3, 6, and X_5 _contain the NOR regions. Certain chromosomes have specific regions represented by colored bars on the left of the chromosomes, 'w' means that the region is relatively under-represented (see text). The numbers on the right refer to platypus chromosome paints that hybridized to the indicated regions. Middle: the pairing regions of the nine sex chromosomes determined by chromosome painting on mitotic preparations. Those of Y_3 _with X_3 _could not be determined in mitotic metaphases. Bottom: G-banded female karyotype of *T. aculeatus*. The female has 32 chromosome pairs and no unpaired chromosomes.

**Figure 3:**
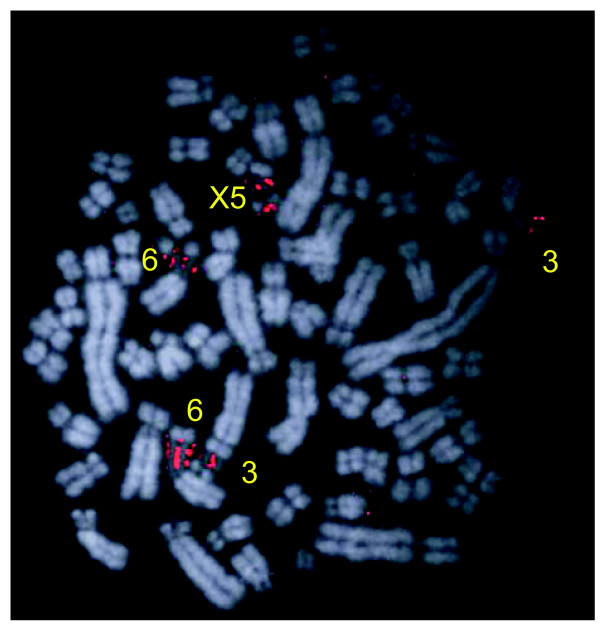
The NOR regions of *T. aculeatus*. FISH with a 28S specific probe was used for identification.

The chromosome paints were used to identify autosome pairs and sex chromosomes in male and female echidnas. Figure 2 shows a G-banded karyotype of male (upper) and female (lower) echidna chromosomes arranged in size and identified by the chromosome painting results (see below). The upper part of both the male and female karyotypes show 27 pairs of autosomes that should form bivalents at meiosis, and the lower parts show the nine unpaired sex-chromosomes in male and five paired sex-chromosomes in female. The paired autosomes can be divided into a group that contains submetacentric chromosomes (chromosomes 1 to 8) and a group of smaller metacentric and submetacentric chromosomes. Chromosomes 3, 6 and X_5 _contain nucleolus organizer regions (Figure 2) determined by Ag-NOR (nucleolar organizing region) staining and by FISH using a probe specific for 28S rDNA (Figure 3).

**Figure 4:**
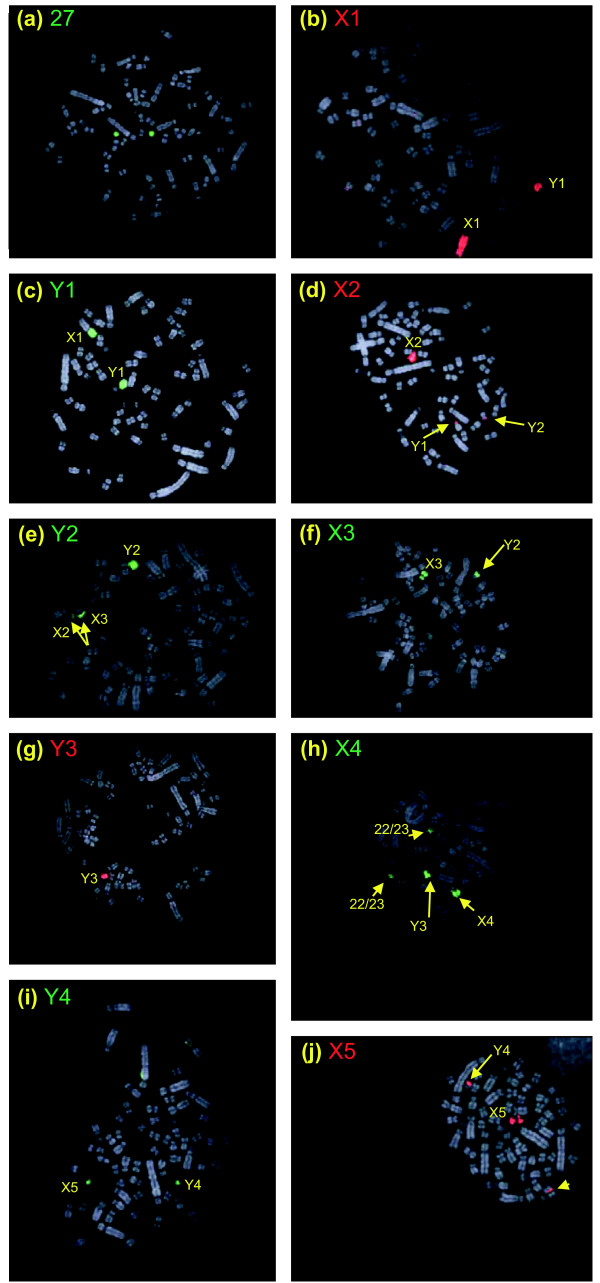
Male *Tac *chromosome identification. **(a) ***Tac *27 is a pair. **(b) **Paint X_1 _identifies X_1 _and Y_1_q. **(c) **Paint Y_1 _covers chromosome Y_1 _and X_1_p. **(d) **Paint X_2 _identifies X_2 _and the region of homology on Y_1 _and Y_2_. **(e) **Paint Y_2 _identifies Y_2 _and the region of homology on X_2 _and X_3_. **(f) **Paint X_3 _covers X_3 _and the pairing region on Y_2_. **(g) **Paint Y_3 _identifies chromosome Y_3_; no homologous regions were observed. **(h) **Paint X_4 _covers X_4_, Y_3 _and a heterochromatic centromeric region on *Oan *22 or 23. **(i) **Paint Y_4 _identifies Y_4 _and the region of homology on X_5_. **(j) **Paint X_5 _covers X_5 _plus the pairing region on Y_4_. The arrow head points to a heterochromatic centromeric region on an autosome.

Special attention was given to chromosome pair 27, as it shows complete homology with platypus X_4 _and partial homology with platypus Y_3 _and Y_4 _(see below), and might, therefore, be an unrecognized sex chromosome. However, both the paints, produced from the two peaks representing the heteromorphic chromosome pair 27, painted both chromosomes 27 totally in both male and female metaphases (Figure 4a). Based on this analysis, the pair was defined as an autosome pair and this was confirmed by analysis of meiotic preparations, which revealed that 27 is not part of the chain.

Chromosome painting in echidna reveals non-specific signals that are present on more than one chromosome pair, indicated by coloured bars next to chromosomes in Figure 2. For instance, when the paint specific for chromosome 1 was hybridized to metaphases, stronger signals were visible on the region indicated on chromosome 1 and a region on chromosome 2. A hybridized chromosome 4 paint gave strong signals on chromosomes 4, 7, and 16 (and others as indicated), weaker signals on X_1 _and Y_1_, and even weaker signals on chromosomes 1 and 2.

The chromosome specificity of these regions was apparent using paints produced by microdissection of these regions. Hybridization to these regions (which hamper identification of chromosomes, and especially pairing regions of the sex chromosomes) was not blocked by pre-hybridization with echidna Cot-1 DNA. To facilitate chromosome identification, an image enhancement procedure was developed to remove these non-specific signals from the image [19]. Molecular characterization of these regions is not considered in this paper.

#### Sex chromosomes in the echidna male

The nine chromosome paints that identified unpaired chromosomes (denoted by X_1_, Y_1_, X_2_, Y_2_, and so on) were used to identify the sex chromosomes of the echidna and predict their order in the meiotic chain. Paint X_1 _hybridized to the whole of chromosome X_1 _and the long arm of Y_1 _(Figure 4b), whereas paint Y_1 _hybridizes to the single chromosome Y_1 _and the short arm of X_1 _(Figure 4c). X_1 _is known to be the first chromosome in the chain [20], and its short arm pairs with the acrocentricY_1_q (second chromosome). The paint X_2 _shows a complete coverage of a single chromosome X_2 _as well as signals on the pairing regions on Y_1_p and Y_2_p (Figure 4d). This result shows that X_2 _is the third and Y_2 _the fourth in the chain. Paint Y_2 _covers Y_2 _and paints the pairing regions on X_2 _and X_3 _(Figure 4e). Paint X_3 _paints the entire chromosome X_3 _as well as the long arm of Y_2 _(Figure 4f), so is the fifth chromosome in the chain. The order of the last four elements is less certain at this stage. Paint Y_3 _covers the tiny chromosome Y_3 _with no signal denoting pairing regions on X_3 _and X_4 _(Figure 4g). Paint X_4 _hybridized to chromosome X_4 _and to Y_3 _and the heterochromatic centromeric regions of chromosome 22 or 23 (Figure 4h). Y_3 _is also painted by chromosome paints 25 and 26, suggesting that it contains shared large non-specific sequences. Paint Y_4 _hybridized to chromosome Y_4 _and to X_5 _p (Figure 4i). Paint X_5 _paints the whole X_5 _and the long arm of Y_4 _(Figure 4j); it also showed hybridization to a heterochromatic centromeric region on an autosome.

#### Sex chromosomes in the echidna female

**Figure 5:**
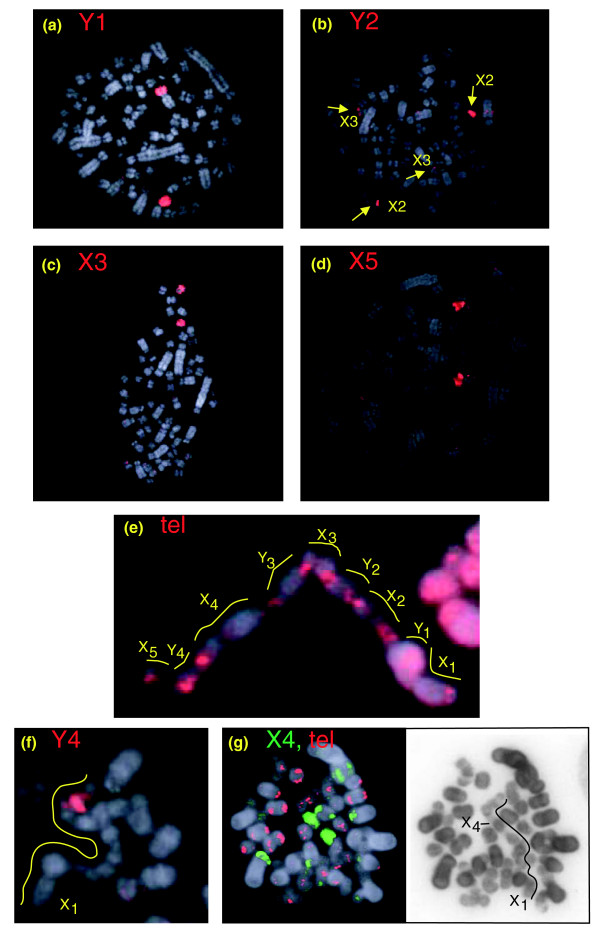
Female *Tac *chromosome identification. **(a) **Paint Y_1 _covers two copies of X_1_p, chromosome Y_1 _is not present. **(b) **Paint Y_2 _covers the homology regions on two copies of X_2 _and X_3_; Y_2 _is not present. **(c) **Paint X_3 _hybridizes to the chromosome pair X_3_. **(d) **Paint X_5 _hybridized to the chromosome pair X_5_. **(e) ***Tac *meiotic chain configuration. Hybridization with telomeric probe confirms a chain of nine elements. **(f) **Paint Y_4 _identifies the last but one chromosome in the chain. **(g) **Paint X_4 _covers X_4 _and Y_3_, the seventh and sixth element of the chain, the chain configuration is at the right.

The same set of nine sex chromosome paints was hybridized to female metaphases to verify which element is an X-chromosome (defined as having one copy in the male and two copies in the female) and which element is a Y-chromosome (one copy in the male and absent in the female). Figure 5 shows some examples of these hybridizations. For instance, chromosome paint Y_2 _hybridized to the pairing regions of X_2 _and X_3_, but identified no copy of the male-specificY_2 _(Figure 5b). The results show that indeed X_1_-X_5 _are X chromosomes and Y_1_-Y_4 _are Y chromosomes. These results also clarified the order of the alternating X and Y chromosomes.

Homologous regions between adjacent X and Y chromosomes are, therefore, demonstrated for all members of the chain of nine except between the small X_3_, Y_3 _and X_4_. These pairing regions all include the distal end of one chromosome arm and do not cross the centromere of the unpaired chromosome. The order of the first five chromosomes of the chain can be deduced from the homology relationships as X_1_Y_1_X_2_Y_2_X_3_. However, the order of the last two X and two Y chromosomes is uncertain by chromosome painting on echidna mitoses, as the pairing regions are too small to detect. However, the results of cross-species painting (see below) and our analyses of chromosome painting of meiotic chains (Figure 5e–g) revealed that X_4 _is the seventh element in the chain (Figure 5g), confirming the order shown in Figure 2.

### Genome wide comparison between echidna and platypus

**Figure 6:**
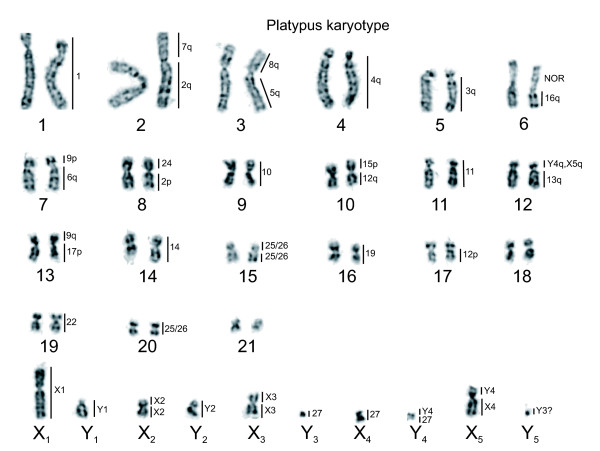
G-banded karyotype of *O. anatinus*. The numbers on the right refer to echidna chromosome paints that hybridized to the indicated regions; compare with Figure 2 top.

Cross-species chromosome painting was used to define chromosome regions conserved between *Oan *and *Tac *and to identify rearrangements that differentiate the karyotypes of the two species. Figures 2a and 6 show the *Oan *and *Tac *homology maps, with the homologous regions indicated on the right of each chromosome.

#### Comparison of platypus-echidna autosomes

**Figure 7:**
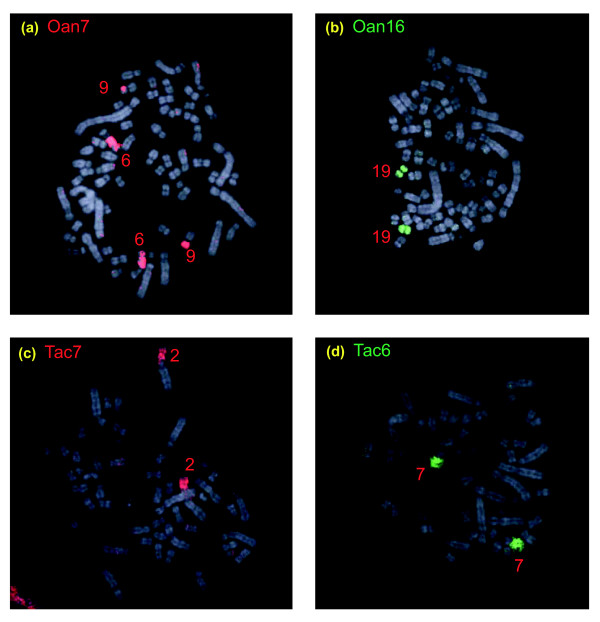
Examples of cross-species chromosome painting to autosomes. **(a) **Paint *Oan *7 and **(b) **paint *Oan *16 hybridized to *Tac *6q and 9p, and *Tac *19, respectively. **(c) **Paint *Tac *7 and **(d) **paint *Tac *6 hybridized to *Oan *2p and *Oan *7q (reverse of 6a), respectively. Note that *Tac *6 is a NOR-bearing chromosome but *Oan *7 is not.

Cross-species painting showed that entire *Oan *chromosomes 1, 4, 5, 9, 11, 14, 16, and 19 are conserved in the *Tac *karyotype as chromosomes 1, 4, 3, 10, 11, 14, 19, and 22, respectively (Figures 2a, 6 and 7b). *Oan *chromosomes 15 and 20 are conserved on either *Tac *25 or 26, which are similar in size and could not be separated by flow sorting.

Several platypus chromosomes (*Oan *2, 3, 7, 8, 10, 12, 13) paint two echidna chromosomes or chromosome arms, representing a centric fusion in the platypus lineage, or a fission in the echidna lineage (Figures 2a and 7a,d). *Vice versa*, there are also four echidna chromosomes (*Tac *2, 7, 9, 12) each of whose arms are homologous to two platypus chromosomes, implying either fissions in the platypus lineage or fusions in the echidna lineage (Figures 6 and 7c). It is not possible to distinguish centric fusions from centric fissions without reference to outgroup species.

*Tac *homologous regions for *Oan *18, 17p and 21 could not be determined, probably because these regions are homologous to *Tac *chromosomes with large amounts of 'non-chromosome-specific' repetitive DNA (see first section of results). Similarly, homologous regions were not determined for short regions on *Tac *3p, 4p, 5p, 6p, 8p, 13p, 15q, 16p, 17q, 18, 20, 21, 23, 24p and the large regions on 1q and 2q. These blocks correspond to specific *Tac *regions identified by paints made from the equivalent regions by microdissection (depicted in different colors at the left in Figure 2 top).

The NOR-bearing regions are not on homologous chromosomes in the platypus and echidna. In platypus, *Oan *6 contains the NOR region; this chromosome is homologous to *Tac *16. In echidna, *Tac *chromosomes 3, 6, and X_5 _are the NOR bearing chromosomes (Figure 3); these chromosomes are homologous to *Oan *5, 7, and 12p.

#### Comparison of platypus and echidna sex chromosomes

**Figure 8:**
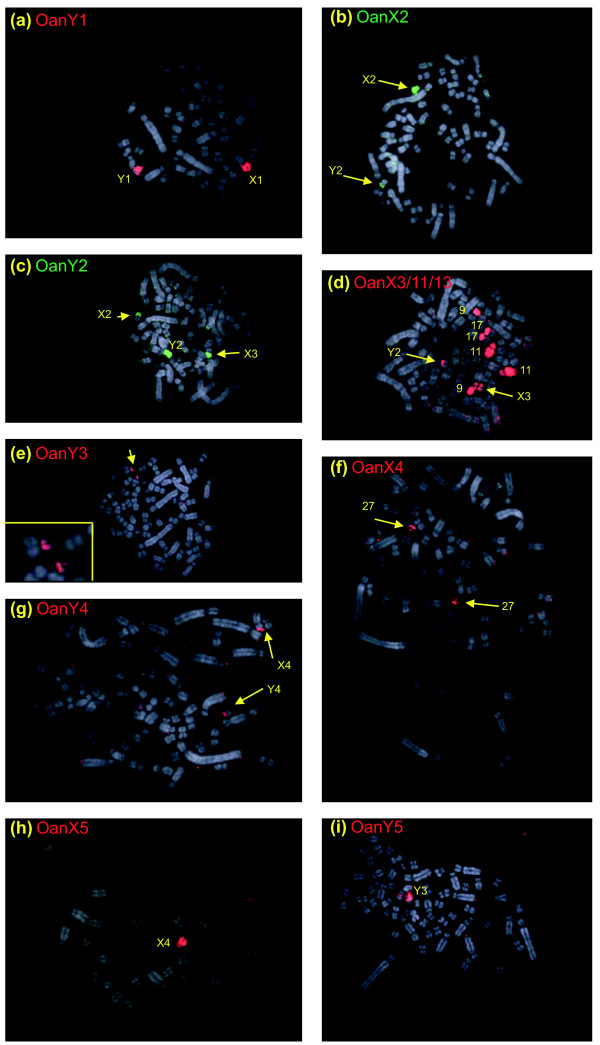
*Oan *chromosome paints hybridized to male *Tac *metaphases. **(a) **Paint *Oan *Y_1 _covers *Tac *Y_1 _and *Tac *X_1_p. **(b) **Paint *Oan *X_2 _hybridizes to *Tac *X_2 _and the pairing region on *Tac *Y_2_. **(c) **Paint *Oan *Y_2 _covers *Tac *Y_2 _and hybridizes to the pairing regions on *Tac *X_2 _and *Tac *X_3_. **(d) **Paint *Oan *X_3 _is mixed with paint for 11 and 13 (see text). As well as to other chromosomes as indicated, *Tac *X_3 _and the pairing region on *Tac *Y_2 _are painted. **(e) **Paint *Oan *Y_3 _hybridizes to a region of *Tac *27 (see inset). **(f) **Paint *Oan *X_4 _hybridizes to *Tac *27. **(g) **Paint *Oan *Y_4 _hybridizes to the top of *Tac *X_4 _and a region on *Tac *Y_4_. **(h) **Paint *Oan *X_5 _covers *Tac *X_4_. **(i) **Paint *Oan *Y_5 _hybridized to *Tac *Y_3_.

**Figure 9:**
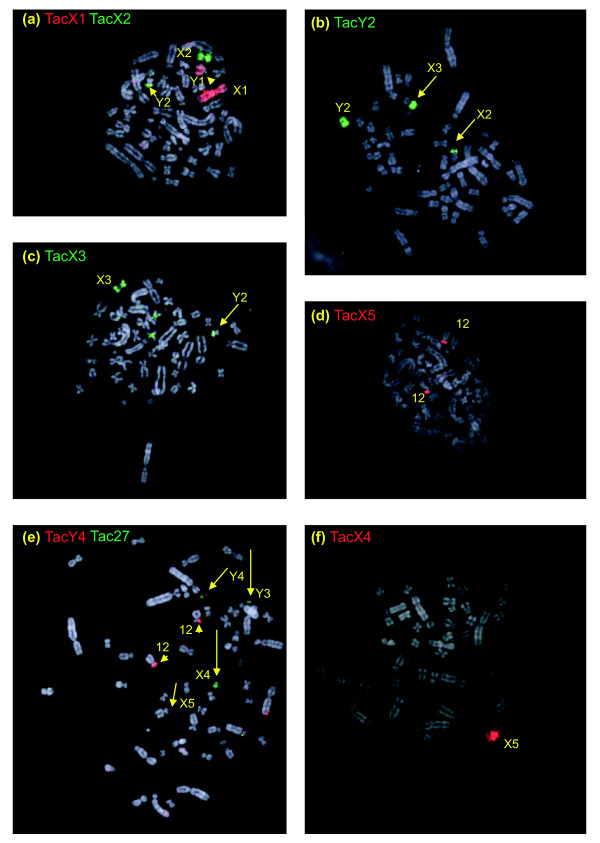
*Tac *chromosome paints hybridized to male *Oan *metaphases. **(a) **Paint *Tac *X_1 _(red) hybridized to *Oan *X_1 _and *Oan *Y_1_q. Paint *Tac *X_2 _(green) hybridized to *Oan *X_2 _and the pairing region on *Oan *Y_2 _and Y_1_p (arrow head). **(b) **Paint *Tac *Y_2 _covers *Oan *Y_2 _and hybridized to *Oan *X_2 _and *Oan *X_3_. **(c) **Paint *Tac *X_3 _hybridized to *Oan *X_3 _and the pairing region on *Oan *Y_2_, the two additional signals are centromeric heterochromatin. **(d) ***Tac *paint X_5 _hybridized to *Oan *12p. **(e) **Paint *Tac *Y_4 _(red) hybridizes to *Oan *12p, and to *Oan *X_5_p and *Oan *Y_4_p. Paint *Tac *27 identified *Oan *Y_3_, *Oan *X_4_, and *Oan *Y_4_q. **(f) **Paint *Tac *X_4 _covers *Oan *X_5_.

**Figure 10:**
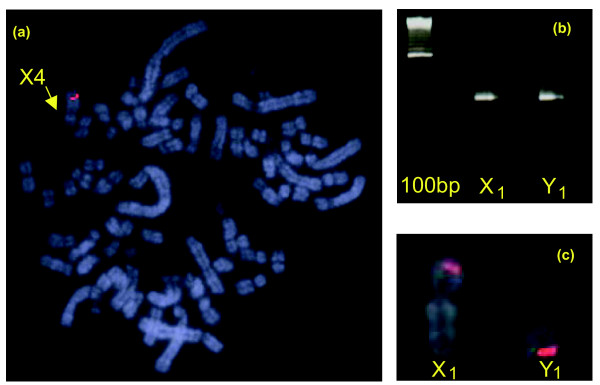
Gene mapping. **(a) **BAC-clone FISH of *DMRT1 *on echidna X_4_. **(b) **Localization of *PDE6A *by PCR. **(c) **BAC-clone FISH mapping *PDE6A *to the pairing regions of X_1 _and Y_1_.

Cross-species painting with *Oan *and *Tac *X-Y probes shows that *Oan *X_1_, Y_1_, X_2_, Y_2_, X_3 _are homologous to *Tac *X_1_, Y_1_, X_2_, Y_2_, and X_3_, respectively (Figures 8a–d and 9a–c), but one X chromosome in each and homologous regions of the flanking Y chromosomes are non-homologous. Reciprocal chromosome painting shows that *Oan *X_5 _and *Tac *X_4 _are homologous. The *Oan *X_5 _paint hybridized to *Tac *X_4 _(Figure 8h), the *Tac *X_4 _paint hybridized to *Oan *X_5 _(Figure 9f); neither hybridization detected pairing regions in adjacent Y chromosomes. Confirmation that the large *Tac *X_4 _chromosome is the genetic homologue of the *Oan *X_5 _is provided by the assignment of the *DMRT1 *gene complex to *Tac *X_4_q (Figure 10a).

An important result was that *Oan *Y_3 _and X_4 _paints hybridized to a *Tac *autosome, and that *Tac *X_5 _paint hybridized to an *Oan *autosome. *Oan *paint X_4 _hybridized to the whole *Tac *27 (Figure 8f). *Oan *paint Y_3 _hybridized to a small region of *Tac *chromosome 27 (Figure 8e), confirmed as an autosome (Figure 4a). *Oan *paint Y_4 _hybridized to *Tac *Y_4_p and the distal end of *Tac *X_4_p (Figure 8g). Reciprocally, *Tac *X_5 _paint hybridized to platypus chromosome 12p (Figure 9d). *Tac *paint Y_4 _hybridized to four regions: the two homologues *Oan *12p, *Oan *X_5_p and *Oan *Y_4_p and *Tac *27 identified *Oan *Y_3_, *Oan *X_4_, and *Oan *Y_4_q (Figure 9e). Many metaphases were observed to confirm these signals. We conclude that *Tac *Y_4_q and X_5 _represent autosomes in platypus and parts of *Oan *Y_3_, X_4 _and Y_4_q are homologous to autosomal regions in echidna.

In the echidna, there are only four Y chromosomes, compared to five in the platypus, suggesting that the small Y_5 _in platypus has been completely lost in the echidna lineage [21]. A surprising result, therefore, was the hybridization of the *Oan *Y_5 _paint to *Tac *Y_3_, a strong indication that *Oan *Y_5 _is represented in the echidna chromosome chain of nine (Figure 8i). No reliable signals on chromosomes other than *Tac *Y_3 _were observed. Attempts to hybridize the chromosome paint of *Tac *Y_3 _paint onto *Oan *male metaphases produced no reliable signal.

### Mapping chicken-human homologous genes

**Figure 11:**
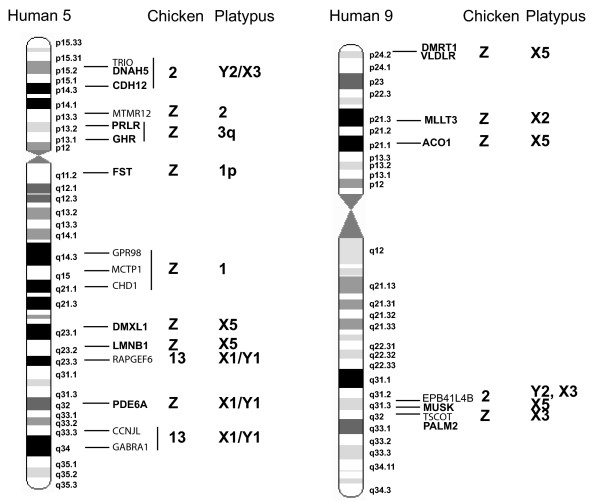
Location of mapped human 5 and human 9 genes in human, chicken and platypus. Gene names in italic are mapped in platypus by PCR, gene names in bold are mapped by both PCR and BAC-clone FISH. EPB41L4B is in contig 29 (Additional data file 1, *P15RS*).

**Figure 12:**
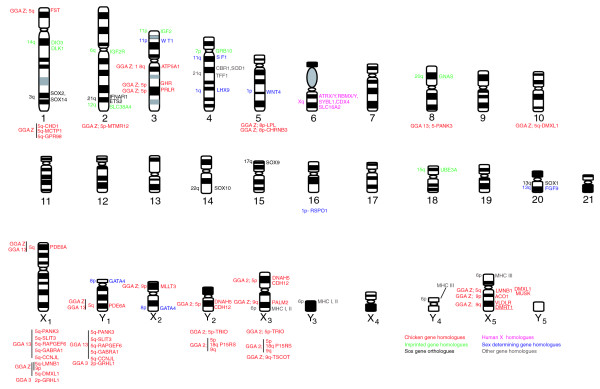
Idiogram showing location of genes in platypus. Gene names in pink are human X-linked genes, gene names in green are homologues of genes imprinted in mouse, gene names in blue are homologues of genes in the mammalian sex determining pathway, gene names in black are Sox gene orthologues, and genes in grey are other previously mapped genes. Gene names in red under a chromosome are mapped in this report by PCR only. Gene names in red next to a chromosome are mapped in this report by PCR and BAC-clone FISH (*DMRT1 *mapped previously [6,8]). The numbers on the left refer to the gene location in human. The location in chicken is indicated as well, for example, FST located on platypus 1p is on human 5q, chicken Z.

In order to test the hypothesis that the bird Z chromosome is represented in the monotreme sex chromosome chain, platypus homologues of nineteen chicken-Z genes (together with chicken 2, 3, and 13 genes) were mapped to platypus chromosomes by two independent methods, PCR amplification of chromosome specific DNA and FISH localization of platypus bacterial artificial chromosome (BACs). Gene mapping results are summarized in Figures 11 and 12, and see Additional data file 1. This file also shows platypus contigs that contain the mapped and predicted genes extending the regions of homology.

Gene loci in chicken are designated in the following section according to their established conserved synteny with human chromosomes. For instance, chicken Z is homologous with regions of human chromosomes 5, 9, 8 and 18 and chicken chromosome 2, 3 and 13 also share homology with human chromosome 5 [9]. Figure 12 presents a standard idiogram, based on G-banding and chromosome painting, on which the present and previous [10,11,22-25] gene assignments are shown. Consideration of the contigs in Ensembl to which these genes belong greatly expands the size of the syntenic regions (Additional data file 1). Most of the conserved syntenies that we have observed between platypus chromosomes and chicken Z are in five groups.

The first group includes platypus homologues of chicken Z and human 9 (*Gallus gallus *(GGA)-Z/*Homo sapiens *(HAS)-9) genes. Three genes mapped to platypus X_5_, one mapped to X_2 _and two mapped to X_3 _(Figure 12). Consideration of the contigs to which these genes belong greatly expands the size of the homologous regions (Additional data file 1).

The second group includes platypus homologues of chicken Z and human 5 (GGA-Z/HSA-5) genes. Ten genes are distributed over platypus chromosomes 1, 2, 3 and the sex chromosomes. Only one GGA-Z/HSA-5 gene (*PDE6A*) mapped to platypus X_1 _by both PCR and BAC-clone mapping. This gene mapped on the pairing region of X_1_p and Y_1_q, distal to the centromere on each (Figure 10b,c), and is contained in platypus contig 269 with nine other genes, four of which are homologous to GGA-13/HSA-5q (Additional data file 1). Two platypus genes (*LMNB1 *and *DMXL1*) homologous to chicken Z and human 5 (GGA-Z/HSA-5) mapped to platypus X_5 _by both PCR and BAC-clone mapping. The homologue of the chicken gene *LMNB1 *PCR-mapped also to X_1 _but not to Y_1_, suggesting that the PCR product of X_1 _may represent a section of the X_5 _gene that has a copy (paralogue) on X_1_. Likewise, *DMXL1 *was PCR-mapped also to *Oan *10 as well as X_1 _(but not Y_1_), again indicating that a section of the *DMXL1*-X_5 _gene has a copy (paralogue) on both *Oan *10 and X_1_. The seven other GGA-Z/HSA 5 genes map to *Oan *1, 2 and 3 by PCR or BAC-FISH.

The third group includes platypus homologues of chicken 13 and human 5 (GGA-13/HSA-5), and chicken 3 and human 2 (GGA-3/HSA-2) genes. Five GGA-13/HSA-5 genes and one GGA-3/HSA-2 gene mapped to the pairing regions of X_1 _and Y_1 _(Figures 11 and 12). *PANK3 *also PCR-mapped to *Oan *8, which might be a paralogue. Thus, the three GGA-Z/HSA-5 homologues on X_1 _are accompanied by GGA-13/HSA-5 genes and one GGA-3/HSA-2 gene. The majority of genes in the respective contigs (10, 269, 847, 127) are GGA-13/HSA-5 genes, one is a GGA-3/HSA-8 gene and three are GGA-3/HSA-2 genes.

The fourth group includes platypus homologues of chicken 2 and human 5 (GGA-2/HSA-5), and chicken 2 and human 18 (GGA-2/HSA-18) genes. Three GGA-2/HSA-5 genes mapped to *Oan *Y_2 _and X_3_. These three genes are accompanied by the GGA-2/HSA-18 gene *P15RS*. This gene is in contig 29, which contains 7 GGA-2/HSA-5 genes, 3 GGA-2/HSA-9 genes and 3 GGA-2/HSA-18 genes.

The fifth group includes platypus homologues of chicken Z and human 8 (GGA-Z/HSA-8), and chicken Z and human 18 (GGA-Z/HSA-18) genes. Two GGA-Z/HSA-8 genes mapped to platypus chromosome 5, and one GGA-Z/HSA-18 gene mapped to platypus chromosome 3.

Thus, mapping platypus homologues of chicken Z genes revealed homology not only with platypus X_5 _but also with platypus X_1_, Y_1_, X_2 _and X_3_. Other genes from human chromosomes 5 and 9, which are not homologous to the chicken Z, also mapped to platypus sex chromosomes. A set of GGA-Z genes homologous to HSA-5, 8 or 18 (see above), but so far not HSA-9, mapped to platypus autosomes. So far, no chicken Z genes have been mapped to platypus X_4_, but this may not be surprising as X_4 _is homologous to an echidna autosome.

A selection of these genes was PCR-mapped to echidna chromosome-specific DNA using the same primers. *GPR98 *(*Oan *1) mapped to *Tac *1, *GRHL1 *(*Oan *X1, Y1) mapped to *Tac *X_1 _but not Y_1_, *RAPGEF6 *(*Oan *X_1_, Y_1_) mapped to *Tac *X_1 _and Y_1_. *MLLT3 *(*Oan *X_2_) mapped to *Tac *X_2_, *TRIO *(*Oan *Y_2_, X_3_) mapped to *Tac *Y_2 _and X_3_, *TSCOT (Oan *X_3_) mapped to *Tac *X_3_, and *LMNB1 *(*Oan *X_1_, X_5_) mapped to *Tac *X_4_. *DMRT1 *(Oan X_5_) mapped to Tac X_4 _by BAC-FISH (Figure 10a). These results confirm homologies between the platypus and echidna chromosomes determined by chromosome painting.

## Discussion

We confirm previous cytological studies of echidna mitotic and meiotic chromosomes that showed that the male short-beaked echidna has 63 chromosomes - 27 pairs of autosomes and 9 sex chromosomes [20,21,26] - and establish that the sex chromosome constitution is 5 Xs and 4 Ys. These chromosomes form a chain of nine at meiosis, expected (by analogy to platypus) to be in an alternating X-Y order. Adjacent members are expected to be held together by pairing within 16 pseudoautosomal regions (one per chromosome arm except for X_1_q and X_5_p, which terminate the chain). Each sex chromosome and 13 of the expected 16 pairing regions of the 5 Xs and 4 Ys (Figure 2, middle) were identified in this study. The alternating order in the chain is directly confirmed by painting in meiosis I metaphases (Figure 5e–g).

### Homology between platypus and echidna chromosomes

Ten chromosomes are completely conserved between echidna and platypus. The non-conserved chromosomes differ between the two species by centric rearrangements only, which are charted in Figures 2 and 6. Without comparative data from an outgroup it is not possible to distinguish fusions in one lineage from fissions in the other.

Both platypus and echidna have prominent NORs on the short arm of chromosome 6, which was thought to be homologous, because of their similar size and morphology [21]. However, chromosome painting shows that platypus chromosome 6 is homologous to echidna chromosome16. In addition, echidna has NORs on X_4 _and chromosome 3. Non-homology of NOR-bearing chromosomes is not surprising in view of their rearrangement in many closely related species.

### Differences between platypus and echidna sex chromosome chains

The constitution of the sex chromosome system in *Tac *differs from that in *Oan *in their number, order and in the identity of one XY pair. Firstly, chromosome painting shows that there are five Xs and five Ys in platypus but five Xs and only four Ys in the echidna. The missing platypus Y_5 _is a very small chromosome, and it had been previously supposed to have been lost from the echidna lineage [21,27]. However, the presence of a strongly hybridizing region on the larger echidna Y_3 _using the platypus Y_5 _paint suggests that the content of this platypus Y is incorporated into echidnaY_3_. The surprising finding is that these two Y-chromosomes are at different locations in the chain. Secondly, *Oan *X_5 _and *Tac *X_4 _are shown to be homologous chromosomes by chromosome painting, and by sharing the *DMRT1 *gene cluster (Figure 10a). However, they occupy different positions in the chain. Thirdly, the most telling result we obtained was the finding that the platypus and echidna sex chromosome chains contain elements (*Oan *chromosomes Y_3_, X_4 _and Y_4_, and *Tac *X_5 _and Y_4_) that are not homologous; platypus X_4 _paints an autosome (chromosome 27) in echidna and echidna X_5 _paints an autosome (chromosome 12) in platypus.

Thus, the chain in these monotreme species differs in both order and constitution, indicating that the chain continued to evolve after the divergence of platypus and echidna approximately 25 MYA [28]. One can speculate that the primary sex determining locus is more likely to be found on the non-pairing regions of the X or Y chromosomes that are shared between platypus and echidna rather than on those that are autosomal in either.

Although the comparative painting reported here is a genome-wide comparison between the two monotreme species, it cannot, of course, reveal the gene content of the chromosomes, which requires comparative gene mapping.

### Mapping chicken-human homologous genes

Comparative gene mapping was used to establish that homologous genes are together in the same contiguous region in both species indicating chromosome homology. This does not exclude the possibility of non-orthology for some of the contiguous genes, but the likelihood of multiple, independent exceptional events is reduced as more homologues are discovered in the region. As the chance of independent evolution of syntenic regions is reduced, the likelihood of shared descent from the same chromosomal region in a common ancestor is increased.

The comparative mapping results show that monotreme sex chromosomes contain genes homologous to the chicken Z chromosome and chicken autosomes, with the implication that the sex determining system might be related to an ancestral sauropsid system. The early finding of the *DMRT1 *gene on platypus X_5 _prompted our search for other chicken Z genes on this chromosome by mapping homologues of GGA-Z/HSA-9 genes followed by homologues of GGA-Z/HSA-5 genes. This led to the observation of chicken Z and autosomal homologues on other platypus sex chromosomes.

With regard to platypus X_1_, the preliminary results of the draft genome sequence of the female platypus (Ensembl release 44) and a recent comparative mapping study of therian X-linked genes (F Veyrunes, personal communication) show that platypus X_1 _does not share homology with the therian X as previously reported [14-18]. X-linked genes on human Xq assigned by FISH localization of BACs so far all map to platypus chromosome 6 [10]. These on human Xp map to platypus chromosome 15 and 18 [23] (F Veyrunes, personal communication). The results presented here reveal instead that platypus X_1 _shares homology with chicken chromosomes 3, 13, and Z and the corresponding human chromosomes 2, 5, 8, and 9 (Additional data file 1). Thus, our mapping data show that X_1 _shares homology with those chicken chromosomes that are homologous to human autosomes.

The implications of some of the platypus gene assignments require further discussion. For example, the motivation to map *RAPGEF6 *was that this gene is close to the chicken Z *HINT1 *homologue in human (HSA-5q), although on a different chromosome in chicken (GGA-13). *HINT1 *might be involved in the chicken sex determining pathway, so may be a candidate for the primary sex determining locus in platypus; mapping a *HINT1 *homologue was unsuccessful. However, the localization of *RAPGEF6 *to *Oan *X_1_, Y_1 _makes it unlikely that this region contains the primary sex determinant. Note that platypus X_1_p and Y_1_q contain homologues of genes on chicken 3, 13 and Z (mapped in this report). The chicken 13 and the chicken Z regions were presumably syntenic before the divergence of Prototheria and Theria as these regions are syntenic in both monotremes and human.

The platypus homologues that map to X_2 _(*MLLT3*), X_3 _(*TSCOT*, *PALM2*), and both X_3 _and Y_2 _(*CDH12*, *DNAH5*, *TRIO*, *P15RS*) assign their respective contigs to these sex chromosomes (Figure 12 and Additional data file 1), indicating that parts of chicken 2 and chicken Z were fused before the divergence of Prototheria and Theria, and that the fission of chicken chromosome 2 into the regions on human chromosome 5, 9, and 18 occurred after the divergence of Prototheria and Theria.

Previous work showed that platypus chromosome X_5 _contains the *DMRT1-2-3 *complex, whose homologue maps to the Z chromosome in chicken, and to human chromosome 9 [6,8]. We show here that several other genes with homologues on the chicken Z also map to the platypus X_5_. Identification of the contigs that include these genes maps a large fraction of the chicken Z to platypus X_5_, indicating that a large region of human 9 homologous to chicken Z is conserved on platypus chromosome X_5_. Platypus major histocompability complex (MHC) class III genes have been mapped recently to the pairing segments of X_5 _and Y_4 _and MHC class I and II genes have been mapped to the pairing regions of X_3 _and Y_3 _[29]. We note that platypus X_4 _(homologous to the echidna autosome *Tac *27) separates these two clusters, while in echidna the homologues of platypus X_5 _and Y_4 _(that is, *Tac *X_4 _and Y_4_) that carry the MHC genes are adjacent to X_3 _and Y_3_, suggesting that the insertion of X_4 _in platypus was a later event in the evolution of the monotreme meiotic chain. So far, no homologues of chicken Z map to platypus X_4_, possibly because it is homologous to an echidna autosome.

The gene mapping results described here assign several genes to the pairing regions of platypus Y chromosomes. The sequence reported in Ensembl does not provide this information as it is based on the female genome, so Y-linked genes are not included.

Not all chicken Z homologous genes are located on the monotreme sex chromosomes. Seven additional GGA-Z/HSA-5 genes map to platypus autosomes 1, 2, or 3 (Figures 11 and 12). The GGA-Z/HSA-8 genes *LPL *and *CHRNB3 *map to platypus chromosome 5 and the GGA-Z/HSA-18 gene *ATP5A1 *maps to platypus chromosome 3. Platypus chromosome 3 contains homologues of genes localized on chicken Z and human 5 and 18. This means that these two human regions were syntenic before monotreme-therian divergence and became separated only in the mammalian lineage after this divergence.

The monotreme regions homologous to chicken Z are considerably rearranged and distributed over autosomes and at least four X chromosomes and the corresponding Ys. The (single) Z homologues on X_1_, Y_1 _and X_2 _are accompanied by other non-Z homologues in their respective contigs. X_3 _seems to have a large region homologous to chicken Z as it contains contig 278 with a size around 0.8 Mb. This contig has chicken Z genes that are homologous only to human chromosome 9 and 5. In Ensembl release 45, contig 278 is part of ultracontig 84 with a size of around 8 Mb containing more chicken Z (human 5 and 9) homologous genes. Only chicken Z genes have so far been mapped to platypus X_5 _and most of these are homologous to human 9 and a few to human 5. As both X_3 _and X_5 _contain human 5 and 9 genes (that are homologous to chicken Z), the separation into these human 5 and human 9 regions must have occurred later in the therian lineage. Finally, the platypus autosomes 1, 2 and 3 also contain chicken Z genes, suggesting that these chromosomes may represent the partners in original translocations involving a putative ancestral Z chromosome.

The results shown in Figure 12 all confirmed the homologies found by chromosome painting in this report and in our previous paper [7]. In particular, a number of FISH assignments confirmed the pairing regions between X and Y chromosomes. These early mapping results should help in the identification of the primary sex-determining locus, in the investigation of mechanisms of dosage compensation and in understanding the evolution of vertebrate sex chromosomes.

An exchange mechanism for the evolution of the multiple sex chromosomes of the platypus was postulated previously [7,21]. The chain development was suggested to start with an ancestral pair of differentiated sex chromosomes, one of which was repeatedly involved in exchanges with autosomes. An alternative view [27,30] suggests that the chain arose as the result of hybridization between two ancestral monotreme populations, each with a different set of Robertsonian translocations resulting in a male heterozygous for unpaired sex chromosomes. Common to all models is that the rearranged autosomes in the chain evolved into Y chromosomes. Our finding that different rearrangements occurred in the two monotreme lineages after the platypus-echidna divergence (25 MYA [28]) is easier to reconcile with a model of successive translocation, rather than the unlikely alternative of additional hybridizations between populations differing in other Robertsonian rearrangements.

## Conclusion

Our cross-species painting studies of the monotreme sex chromosome complements shows that the platypus and echidna translocation chains share homology over four of the five X chromosomes, but one in each species is entirely non-homologous. This means that the chains continued to evolve after the divergence of platypus and echidna.

Our comparative mapping studies show chicken Z homologous genes in the sex chromosome system with the main clusters on platypus X_3 _and X_5 _and echidna X_3 _and X_4_. Other Z homologous genes map to autosomes, indicating many rearrangements between the monotreme and avian lineages.

In combination with the mapping data available in current Ensembl release 44, our results also reveal homology of platypus X_1 _to chicken 3, 13, Z, 11, and 12, which are homologous to human autosomes. This suggests that the monotreme's XY chromosome system is unrelated to the therian XY system. This is further explored by F Veyrunes (personal communication) in comparative studies with therian X-linked genes, and it may mean that the therian XY system evolved after the prototherian and therian divergence, but before the divergence of marsupials, and is, therefore, younger than previously anticipated [31].

It is important to note that three monotreme X chromosomes have large differential regions. The differential region on platypus X_5 _(echidna X_4_) is homologous to chicken Z, that of X_3 _is homologous to chicken 2 and Z, and the large differential region on X_1 _seems mostly homologous to chicken 3 and 12. These differential regions are completely different from those of the therian X and Y chromosomes, indicating again that the monotreme and therian sex chromosome systems have different origins. We believe that the comparative mapping results reported here will be useful in the continuing search for the monotreme sex determining switch, and in future studies on sex chromosome evolution and dosage compensation mechanisms.

It will be instructive to extend the genome comparison between birds and monotremes to other amniotes, such as snakes and lizards. These comparisons will enable the construction of the ancestral karyotype of sauropsids and mammals and reveal the chromosome evolutionary events that occurred at the origin of the sauropsid and mammalian lineages.

## Materials and methods

### Chromosome paint generation

Primary fibroblast cultures from the short-beaked echidna (*T. aculeatus*, 2n = 63 male, 64 female) were established routinely in standard medium at 32°C (AEEC permit no. R.CG.07.03 and AEC permit no S-049-2006, NSW P&W permit S10443). Flow sorting, chromosome paint production and FISH were performed according to the protocol described previously [7,32]. Platypus (2n = 52) chromosome paints and metaphases were generated as previously described for the characterization of the platypus sex chromosome complement [7].

### Cot-1 preparation

Cot-1 DNA was prepared as described [33], except that the renatured (double-stranded) Cot-1 DNA was separated from the digested (single-stranded) DNA using a phenol-chloroform-extraction method. Purified echidna Cot-1 DNA (30 mg) was used for FISH.

### Preparation of meiotic cells

Meiotic cells were obtained from animals captured at the upper Barnard river, New South Wales, Australia during breeding season (AEEC and AEC permits to FG as above). The captured animals were euthanased with an intraperitoneal injection of pentobarbitone sodium (Nembutal, Boehringer Ingelheim, NSW, Australia) at a dose of 0.1 mg/g body weight. Meiotic cells were obtained by disaggregating the testis. The material was either directly fixed in methanol/acetic acid (3:1) or incubated in 0.075 KCl M at 37°C as hypotonic treatment to improve spreading of metaphase cells and then fixed.

### Chromosome microdissection

Microdissection-derived chromosome specific paints of specific blocks were generated as described previously [34]. In short, short-beaked echidna metaphases were dropped onto wet cover slips and subsequently digested with 0.015% trypsin for 1 minute and stained with Giemsa. Microdissected material was collected in a drop containing 10 mM NaCl, 10 mM Tris-HCL, 1 mM EDTA, 0.1% SDS, 0.1% Triton ×100, 1.44 mg/ml Proteinase K (Sigma-Aldrich, Gillingham, Dorset, UK), 30% glycerol. After 1 hour incubation at 60°C this drop was transferred to 5 μl of 1× sequenase buffer (USB, Staufen, Germany), 0.2 mM dNTPs, 5 μM 6 mW primer. A PCR protocol for low temp cycles was next: 5 minutes at 92°C followed by 8 cycles of 2 minutes 20 s at 25°C, 2 minutes at 34°C, 1 minute at 90°C. At the start of each cycle 0.4 units of sequenase (USB) was added. For the high temp cycles 45 μl 1× Buffer D (Invitrogen, Paisley, UK), 0.2 mM dNTP, 5 μM 6 mW primer, 0.3 u SuperTaq polymerase (HT Biotechnology, Cambridge, UK), 0.05% W1 (Sigma) were added. This solution was subjected to 33 cycles of 1 minute at 92°C, 2 minutes at 56°C, 2 minutes at 72°C with a final extension for 5 minutes at 72°C. The DNA was labeled using a standard protocol [32].

### Fluorescence microscopy

Images were captured using the Leica QFISH software (Leica Microsystems, Milton Keynes, UK) and a cooled CCD camera (Photometrics Sensys, Photometrics, Tucson, AZ, USA) mounted on a Leica DMRXA microscope equipped with a 63×, 1.3 NA objective. Cy3, FITC 9 (fluorescein isothiocyanate) and DAPI (4',6-diamidino-2-phenylindole) signals were captured separately as 16 bit black and white images, and merged to a color image. The DAPI image was enhanced with a spatial filter to obtain enhanced chromosome bands. All image processing was performed with Leica CW4000 software.

### Characterization of the short-beaked echidna karyotype

The *Tac *paints produced were hybridized to male (three individuals) and female (one individual) *Tac *metaphase preparations. Multicolor chromosome painting was used to ensure that different peaks represent different chromosomes, to define the order of the unpaired chromosomes and to determine the homologous parts that link these chromosomes in the meiotic chain.

### Mapping chicken-human chromosome 5, 9, 8, 18 homologous genes on platypus and echidna chromosomes

Twenty-nine genes were mapped to platypus chromosomes (Table 1), and eight of these to echidna chromosomes. Two methods, PCR and BAC-clone mapping (indicated by 'a' and 'b' in Table 1) were used to localize the platypus homologues.

**Table 1:** List of gene homologues mapped to platypus chromosomes

Gene	HSA, GGA location	Method*
*LMNB1*: lamin B1	5, Z	a, b
*DMXL1*: DMX like-1	5, Z	a, b
*CHD1*: chromodomain helicase DNA binding protein 1	5, Z	a, b
*MCTP1*: multiple C2 domains, transmembrane 1	5, Z	a
*GPR98*: G protein-coupled receptor 98	5, Z	a
*FST*: follistatin	5, Z	a, b
*GHR*: growth hormone receptor	5, Z	a, b
*MTMR12*: myotubularin related protein 12	5, Z	a
*PDE6A*: rod cGMP-specific 3',5'-cyclic phosphodiesterase alpha-subunit	5, Z	a, b
*PRLR*: prolactin receptor precursor	5, Z	a, b
*GABRA1*: gamma-aminobutyric acid A receptor, alpha 1	5, 13	a
*GHRL1*: grainyhead-like 1 (GRHL1), transcript variant 1	2, 3	a
*SLIT3*: slit homolog 3 protein precursor	5, 13	a
*PANK3*: pantothenate kinase 3	5, 13	a
*CCNJL*: cyclin J-like	5, 13	a
*RAPGEF6*: rap guanine nucleotide exchange factor (GEF) 6	5, 13	a
*CDH12*: cadherin-12 precursor	5, 2	a, b
*DNAH5*: ciliary dynein heavy chain 5	5, 2	a, b
*TRIO*: triple functional domain protein	5, 2	a
*VLDLR*: very low-density lipoprotein receptor	9, Z	a, b
*ACO1*: iron-responsive element-binding protein 1	9, Z	a, b
*MUSK*: muscle-specific kinase receptor	9, Z	a, b
*MLLT3*: protein AF-9	9, Z	a, b
*TSCOT*: thymic stromal cotransporter homolog	9, Z	a
*PALM2*: paralemmin-2	9, Z	b
*P15RS*: P15RS protein	18, 2	a
*LPL*: lipoprotein lipase	8, Z	a
*CHRNB3*: neuronal acetylcholine receptor protein subunit beta-3 precursor	8, Z	a
*ATP5A1*: ATP synthase subunit alpha	18, Z	a, b

*The two methods used to localize the platypus homologues are: a, PCR; b, BAC-clone. See text for details.

#### PCR

A human or chicken exon of the specific gene was blasted to find alignments with the NCBI trace archives of platypus using discontiguous megablast. The alignment was used to design platypus specific primers using PrimerQuest [35]. The primers were used first to amplify pools of chromosome specific DNA, and second to amplify chromosome specific DNAs of the positive pool. The exon was considered to be mapped to a single chromosome if only one pool was positive and only one 'chromosome' in that pool was positive. The size of the PCR product was checked to verify that it was as expected from the primer design section, and the product was sequenced for confirmation.

The sequence of the PCR products was blasted to find alignments with the Ensembl Platypus *Ornithorhynchus anatinus *database release 5. The platypus contigs in the database contain several predicted genes, which were identified by blasting to find alignments with the NCBI human genome database. Homologues of these genes were subsequently localized in chicken by BLAST alignment in Ensembl Chicken.

#### BAC-clone mapping

The above PCR product was used to screen a platypus BAC-clone library (Oa-Bb, Clemson University, South Carolina, USA). Positive clones were labeled by nick translation and positioned on platypus chromosomes by FISH. The presence of the target gene in the BAC clones above was confirmed by sequencing using the same gene specific primers.

### Chromosome homology by comparative gene mapping

Comparative gene mapping was used for the assessment of chromosome homology. It is important to consider whether the homologous genes are likely to be true orthologues. The definition of orthologues is two genes from two different species that derive from a single gene in the last common ancestor of the species [36]. Absolute proof of orthology is difficult on this criterion and was not pursued in this report. Instead, support for chromosome homology was provided when homologous genes were together within the same contiguous region in both species. Instances of non-orthology in the syntenies may exist but the likelihood of multiple, independent exceptional events is reduced when several gene homologues are found in one region.

## Abbreviations

BAC, bacterial artificial chromosome; FISH, fluorescent *in situ *hybridization; GGA, *Gallus gallus*. HSA, *Homo sapiens*. MHC, major histocompability complex; MYA, million years ago; NOR, nucleolar organizing region; *Oan*, *Ornithorhynchus anatinus*; *Tac*, *Tachyglossus aculeatus*.

## Authors' contributions

WR designed and performed most of the experiments and analyzed the data. PCMOB sorted the platypus and echidna chromosomes, FG and ETA undertook the meiotic analysis, OC, DG, VAT, HS, MCW, and FV performed other experiments. JAMG and SJ provided material and JAMG contributed to the writing of the paper. WR and MAFS conceived and supervised the research and wrote the paper.

## Additional data files

The following additional data are available with the on-line version of this paper. Additional data file 1 is a table listing gene assignments to platypus contigs and platypus chromosomes, together with human and chicken locations.

## Supplementary Material

Additional data File 1 Gene assignments to platypus contigs and platypus chromosomes, together with human and chicken locations. Click here for file
