# Supplementary material for: The multiple sex chromosomes of platypus and echidna are not completely identical and several share homology with the avian Z
Source: Genome Biol. 2007 Nov 16;8(11):R243. doi: 10.1186/gb-2007-8-11-r243 (PMC2258203; doi:10.1186/gb-2007-8-11-r243)
Supplement: Additional data File 1 — Gene assignments to platypus contigs and platypus chromosomes, together with human and chicken locations. [file gb-2007-8-11-r243-S1.doc]

Additional file 1

| Gene assignment to platypus contigs | | | | |
| --- | --- | --- | --- | --- |
| **Platypus Contig** | **Predicted gene** | **Human homologue** | **Human location** | **Chicken**  **chromosome** |
|  |  |  |  |  |
| X_1_,Y_1_-Contig10 | 425383 | *High E-value* | -- |  |
|  | 468299 | *FBXO25* | 8 p23.3 | 3 |
|  | 44985 | *KIAA1127* | 5 q34 | 13 |
|  | 15264 | *RARS* | 5 q35.1 | 13 |
| * | 18174 | *PANK3* | 5 q34 | 13 |
| * | 425737 | *SLIT3* | 5 q34 | 13 |
|  | 50107 | *CCDC99* | 5 q35.1 | 13 |
|  | 64808 | *DOCK2* | 5 q33.1 | 13 |
|  | 1716 | *LCP2* | 5q 33.1 | 13 |
|  | 58734 | *KCNMB1* | 5 q34 | 13 |
|  | 412914 | *KCNIP1* | 5 q35.1 | 13 |
| * | 50085 | *GABRA1* | 5 q34 | 13 |
|  | 11328 | *NPM1* | 5 q35 | 13 |
|  | 42118 | *CSNK1A1* | 5 q35.1 | 13 |
|  | 49825 | *FBXW11* | 5 q35.1 | 13 |
| * | 71845 | *GRHL1* | 2 p25.1 | 3 |
|  | 46973 | *TAF1B* | 2 p25 | 3 |
|  | 6725 | *YWHAQ* | 2 p25.1 | 3 |
| X_1_,Y_1_-Contig269 | 448025 | *PPP2R2B* | 5 q32 | 13 |
|  | 391284 | *SPINK6* | 5 q32 | 13 |
|  | 58562 | *SPINK7* | 5 q33.1 | ? |
|  | 391282 | *High E-value* | -- |  |
|  | 58561 | *High E-value* | -- |  |
|  | 468243 | *FBX038* | 5 q33.1 | ? |
|  | 547185 | *5-HT4* | 5 q32 | 13 |
|  | 70173 | *SLC26A2* | 5 q32 | 13 |
| * | 34195 | *PDE6A* | 5q32 | Z |
|  | Novel 9368 | *PPARGC1B* | 5q32 | 13 |
| X_1_,Y_1_-Contig847 * | 142465 | *RAPGEF6* | 5q23.3 | 13 |
| X_1_,Y_1_-Contig127 | 84320 | *SLU7* | 5q33.3 | 13 |
|  | 58501 | *C1QTNF2* | 5q33.3 | 13 |
| * | Novel 12331 | *CCNJL* | 5q33.3 | 13 |
|  | Novel 12332 | *MST101* | 5q33.3 | 13 |
|  | 15180 | *TTC1* | 5q33.3 | 13 |
|  | 120638 | *ADRA1B* | 5q33.3 | 13 |
|  | 15137 | *IL12B* | 5q33.3 | 13 |
|  | 83997 | *UBLCP1* | 5q33.3 | 13 |
|  | 58499 | *FLJ31951* | ? | ? |
|  | 380920 | *EBF1* | 5q33.3 | 13 |
| X_2_-Contig259 * | 499486 | *MLLT3* | 9p21.3 | Z |
|  | Novel10156 | *PDCD6* | 5p15.33 | 12 |
|  | 412441 | *SDHA* | 5p15.33 | 2 |
|  | 84039 | *CCDC127* | 5p15.33 | 2 |
|  | 156158 | *STATIP1* | 18q12.2 | 2 |
|  | 53636 | *SLC39A6* | 18q12.2 | 2 |
|  | 10164 | *CTNNAL1* | 9q31.3 | 2 |
|  | 17664 | *C9orf6* | 9q31.3 | 2 |
|  | 57637 | *TMEFF1* | 9q31.3 | 2 |
|  | 107836 | *LOC347273* | 9q31.3 | 2 |
|  | 404657 | *LOC389257* | 5p15.33 | 2 |
|  | Novel10173 | *KIAA1909* | 5p15.33 | 11 |
|  | 107841 | *SERPINB2* | 18q21.33 | 2 |
|  | 13310 | *TRIM68* | ? | ? |
|  | 51248 | *BTN3A2* | ? | ? |
|  | 384614 | *AHRR* | 5p15.33 | 12 |
| Y_2_,X_3_-Contig22 | 14867 | *POLS* | 5p15.3 | 2 |
|  | 471877 | *ADCY2* | 5p15.3 | 2 |
|  | 424183 | *LOC134121* | 5p15.3 | ? |
|  | 48845 | *MTRR* | 5p15.3 | 2 |
|  | 49448 | *SEM5A* | 5p15.3 | 2 |
|  | 64806 | *LOC134145* | ? | ? |
|  | 64800 | *CCT5* | 5p15.2 | 2 |
|  | 58312 | *MARCH6* | 5p15.2 | 2 |
|  | 83908 | *CMBL* | 5p15.2 | 2 |
|  | 14872 | *DAP* | 5p15.2 | ? |
|  | 527420 | *CTNND2* | 5p15.2 | 2 |
| * | 49833 | *DNAH5* | 5p15.2 | 2 |
| * | 454076 | *TRIO* | 5p15.2 | 2 |
|  | 58368 | *FAM105A* | 5p15.2 | 2 |
|  | 68553 | *FAM105B* | 5p15.2 | 2 |
|  | 68546 | *ANKH* | 5p15.2 | 2 |
| Y_2_,X_3_-Contig37 | 63688 | *CDH9* | 5p14.1 | 2 |
|  | 44758 | *CDH10* | 5p14.2 | 2 |
| * | 68577 | *CDH12* | 5p14.3 | 2 |
|  | 58342 | *CDH18* | 5p14.3 | 2 |
| Y_2_,X_3_-Contig29 | Novel 1740 | *EPB41L4B* | 9 q31.3 | 2 |
|  | 37743 | *C9orf4* | 9 q31.3 | 2 |
|  | 11138 | *C9orf5* | 9 q31.3 | 2 |
|  | 53650 | *GALNT1* | 18q12.2 | 2 |
|  | 445597 | *?* | ? | ? |
|  | 412692 | *C18orf2* | 18q12.2 | 2 |
| * | 450156 | *P15RS* | 18q12.2 | 2 |
|  | Novel 1747 | *NDUFS6* | 5p15.33 | 2 |
|  | Novel 1748 | *IRX2* | 5p15.33 | 2 |
|  | Novel 1749 | *IRX1* | 5p15.33 | 2 |
|  | 66927 | *ADAMTS16* | 5p15.32 | 2 |
|  | 83823 | *KIAA0947* | 5p15.3 | 2 |
|  | 34527 | *MED10* | 5p15.31 | 2 |
|  | 455338 | *FLJ25076* | 5p15.31 | ? |
| X_3_-Contig278 | 502978 | *KIAA1958* | 9q32 | Z |
|  | 60825 | *SNX30* | 9q32 | Z |
|  | 60811 | *C9orf80* | 9q32 | Z |
| * | Novel0847 | *TSCOT* | 9q32 | Z |
|  | 381968 | *PALM2-AKAP2* | 9q32 | Z |
| * | Novel0849 | *PALM2* | 9q31.3 | Z |
|  | 36114 | *APC* | 5q22.2 | Z |
| X_5_-Contig340 | 37772 | *TXN* | 9 q31.3 | ? |
|  | 85287 | *SEL-OB* | 9 q32 | ? |
|  | 108615 | *SVEP1* | 9 q32 | Z |
| * | 3509 | *MUSK* | 9 q31-q32 | Z |
|  | 523475 | *EDG2* | 9 q31.3 | Z |
| X_5_-Contig231 | 528908 | *GLIS3* | 9 p24.2 | Z |
|  | 9048 | *GLIS3* | 9 p24.2 | Z |
|  | 50794 | *RFX3* | 9 p24.2 | Z |
|  | 50788 | *KIAA0020* | 9 p24.2 | Z |
| * | Novel14993 | *VLDLR* | 9 p24 | Z |
| X_5_-Contig451 * | 133314 | *ACO1* | 9p21.1 | Z |
|  | 11237 | *DDX58* | 9p21.1 | ? |
|  | Novel12129 | *?* | ? | ? |
| X_5_- Contig809 | 60422 | *CDKN2B* | 9p21.3 | ?Z |
|  | 85135 | *High E-value* | -- |  |
| * | Novel 11225 | *LMNB1* | 5q23.2 | Z |
|  | 135192 | *LOC732216* | ? | ? |
| X_1_- Contig1073 * | 134058 | *DMXL1* | 5q23.1 | Z |
| 1*-*Contig802 * | 35650 | *FST* | 5 q11.2 | Z |
|  | 548241 | *MOCS2* | 5 q11.2 | Z |
|  | 454964 | *MOCS2* | 5 q11.2 | Z |
|  | **Novel111203** | *?* | ? | ? |
| 1*-*Contig123 | **29104** | *DHX29* | 5q11.2 | Z |
|  | **14819** | *SKIV2L2* | 5q11.2 | Z |
|  | **79223** | *PPAP2A* | 5q11.2 | Z |
|  | **Novel 4887** | *FLJ90709* | 5q11.2 | Z |
|  | **438327** | *DDX4* | 5q11.2 | Z |
|  | **15206** | *ST8SIA4* | 5q21.1 | Z |
| * | **Novel4891** | *CHD1* | 5q21.1 | Z |
|  | **Novel 4892** | *RGMB* | 5q21.1 | Z |
| 1*-*Contig17 | 522061 | *KIAA0371* | 5q15 | Z |
|  | 67498 | *FAM81B* | 5q15 | Z |
|  | 134650 | *MCTP1* | 5q15 | Z |
|  | 34449 | *ANKRD32* | 5q15 | Z |
|  | 49909 | *BRCTD1* | 5q15 | ? |
|  | 420939 | *C5orf36* | 5q15 | ? |
|  | 49907 | *C5orf21* | 5q15 | Z |
|  | 524469 | *ARRDC3* | 5q14.3 | Z |
| *** | 83882 | *GPR98* | 5q14.3 | Z |
|  | 152088 | *LYSMD3* | 5q14.3 | Z |
|  | 67223 | *CETN3* | 5q14.3 | Z |
|  | 507118 | *MEF2C* | 5q14.3 | Z |
|  | 83856 | *TMEM161B* | 5q14.3 | Z |
| 2-Contig1216 | 49755 | *GOLPH* | 5p13.3 | Z |
| *** | 64649 | *MTMR12* | 5p13.3 | Z |
| 3-Contig521 | 15200 | *RAD1* | 5p13.2 | Z |
|  | 377147 | *BXDC2* | 5p13.2 | Z |
|  | 15204 | *AGXT2* | 5p13.2 | Z |
|  | 15205 | *PRLR* | 5p13.2 | Z |
| 3-Contig16250 | 15024 | *GHR* | 5p12 | Z |
| 3- Contig16769 | 65485 | *ATP5A1* | 18q21.1 | Z |
| 5-Contig75 | 387817 | *PSD3* | 8p22 | ? |
| * | 134106 | *LPL* | 8p21.3 | Z |
|  | 473842 | *?* | ? | ? |
|  | 470782 | *NRG1* | 8p12 | Z |
|  | 87009 | *WRN* | 8p12 | 4 |
|  | Novel5953 | *TEX15* | 8p12 | ? |
| 5-Contig232 | 91652 | *INTS10* | 8p21.3 | 4 |
|  | 104749 | *FNTA* | 8p11.21 | Z |
|  | 116005 | *HOOK3* | 8p11.21 | Z |
|  | 33350 | *THAP1* | 8p11.21 | Z |
|  | 40472 | *CHRNA6* | 8p11.21 | Z |
| * | 73478 | *CHRNB3* | 8p11.21 | Z |
|  | 147686 | *C8orf40* | 8p11.21 | 4 |
|  | 459341 | *SLC20A2* | 8p11.21 | 4 |
|  | 1299 | *VDAC3* | 8p11.21 | 22 |
|  | 453645 | *IKBKB* | 8p11.21 | 22 |
|  | 107872 | *PLAT* | 8p11.21 | 22 |
|  | 3034 | *AP3M2* | 8p11.21 | 22 |
|  | 50658 | *MYST3* | 8p11.21 | 22 |
|  | Novel1720 | *ANK1* | 8p11.21 | 22 |
|  | 62091 | *NKX6-3* | 8p11.21 | 22 |
|  | Novel721 | *AGPAT6* | 8p11.21 | 22 |

‘*’ refers to the mapped homologous gene.

Contig809 is listed in the X_5_ section only although *LMNB1* was PCR-mapped to both X_1_ and X_5_. High E-value means a high probability that the score has arisen by chance from an unrelated sequence, the sequence is, therefore, not identified.
